# Impact of a patient safety leadership program on head nurses and clinical nurses: a quasi-experimental study*

**DOI:** 10.1590/1518-8345.4328.3478

**Published:** 2021-10-29

**Authors:** Jianfei Xie, Siqing Ding, Xiaohong Zhang, Xiaolian Li

**Affiliations:** 1 Central South University, Third Xiangya Hospital, Changsha, China.; 2 Central South University, Xiangya Nursing School, Changsha, China.

**Keywords:** Patient Safety, Leadership, Nursing Administration Research, Nurses, Self-Efficacy, Professional Burnout, Seguridad del Paciente, Liderazgo, Investigación en Administración de Enfermería, Enfermeras y Enfermeros, Autoeficacia, Agotamiento Profesional, Segurança do Paciente, Liderança, Pesquisa em Administração de Enfermagem, Enfermeiras e Enfermeiros, Autoeficácia, Esgotamento Profissional

## Abstract

**Objective:**

to evaluate the impact of a patient safety leadership program on head nurses and clinical nurses in the same nursing unit.

**Method:**

a pre-post quasi-experimental study that included 60 head nurses and 240 clinical nurses was conducted. Only the head nurses received patient safety leadership program training for 12 months. Before and after the training, the General Self-Efficacy Scale was completed by the head nurses, and the General Self-Efficacy Scale, the Hospital Leadership Behavior Assessment Scale, the Safety Behavior Scale and the Maslach Burnout Inventory were completed by the clinical nurses. Descriptive and inferential analyses of the data were performed using absolute and relative frequencies, means and standard deviations, and paired t-tests to assess the effect of the training.

**Results:**

both the head nurses’ and the clinical nurses’ self-efficacy increased significantly (p <0.01) after the training. The leadership behavior of the head nurses and the safety behavior of the clinical nurses also improved significantly (p<0.05). We observed a statistically significant reduction in “emotional exhaustion” and an increase in “personal accomplishment” among the clinical nurses (P<0.001).

**Conclusion:**

the patient safety leadership program had a positive impact on the head nurses’ self-efficacy and leadership behavior and the clinical nurses’ self-efficacy, safety behavior and job burnout.

## Abstract (es)

**Objetivo:**

evaluar el impacto de un programa de liderazgo en seguridad del paciente sobre las enfermeras supervisoras y enfermeras clínicas de la misma unidad de enfermería.

**Método:**

un estudio cuasi-experimental pre-post incluyó a 60 enfermeras supervisoras y 240 enfermeras clínicas. Solo las enfermeras supervisoras recibieron formación en el programa de liderazgo en seguridad del paciente durante 12 meses. Antes y después del programa de formación, las enfermeras supervisoras completaron la Escala de Autoeficacia General mientras que las enfermeras clínicas completaron la Escala de Autoeficacia General, la Escala de Evaluación del Comportamiento de Liderazgo Hospitalario, la Escala de Comportamiento de Seguridad y el Inventario de *Burnout* de Maslach. Se realizaron análisis descriptivos e inferenciales de los datos utilizando frecuencias absolutas y relativas, medias y desviaciones estándares, así como la prueba t pareada para evaluar el efecto del programa de formación.

**Resultados:**

la autoeficacia de las enfermeras supervisoras y enfermeras clínicas aumentó significativamente (p<0,01) después del programa de formación. El comportamiento de liderazgo de las enfermeras supervisoras y el comportamiento de seguridad de las enfermeras clínicas también mejoraron significativamente (p<0,05). Observamos una reducción estadísticamente significativa del “agotamiento emocional” y un aumento de la “realización personal” en las enfermeras clínicas (P<0,001).

**Conclusión:** el programa de liderazgo en seguridad del paciente tuvo un impacto positivo en la autoeficacia y el comportamiento de liderazgo de las enfermeras supervisoras, y en la autoeficacia, el comportamiento de seguridad y el desgaste laboral de las enfermeras clínicas.

## Abstract (pt)

**Objetivo:**

avaliar o impacto de um programa de liderança em segurança do paciente sobre os enfermeiros-chefe e enfermeiros clínicos da mesma unidade de enfermagem.

**Método:**

foi conduzido um estudo quasi-experimental, pré-pós teste, que incluiu 60 enfermeiros-chefe e 240 enfermeiros clínicos. Apenas os enfermeiros-chefe receberam o treinamento do programa de liderança em segurança do paciente por 12 meses. Antes e após o treinamento, a Escala Geral de Autoeficácia foi completada pelos enfermeiros-chefe, e a Escala Geral de Autoeficácia, a Escala de Avaliação do Comportamento de Liderança Hospitalar, a Escala de Comportamento de Segurança e o Inventário de *Burnout* de Maslach foram completadas pelos enfermeiros clínicos. As análises descritivas e inferenciais dos dados foram conduzidas utilizando-se frequências absolutas e relativas, média e desvio padrão, assim como a aplicação do teste t pareado para avaliar o efeito do treinamento.

**Resultados:**

a autoeficácia dos enfermeiros-chefe e a dos enfermeiros clínicos aumentou significativamente (p<0,01) após o treinamento. O comportamento de liderança dos enfermeiros-chefe e o comportamento de segurança dos enfermeiros clínicos também melhorou significativamente (p<0,05). Observou-se uma redução estatisticamente significativa da “exaustão emocional” e aumento da “realização pessoal” nos enfermeiros clínicos (P<0,001).

**Conclusão:**

o programa de liderança em segurança do paciente teve um impacto positivo na autoeficácia e comportamento de liderança dos enfermeiros-chefe e na autoeficácia, comportamento de segurança e esgotamento no trabalho dos enfermeiros clínicos.

## Introduction

As a global health priority, patient safety involves preventing medical errors and avoidable adverse events, protecting patients from harm or injury while receiving health care^(1)^. However, 4 out of 10 patients are harmed in primary and ambulatory care worldwide^(2)^, 134 million adverse events occur each year in hospitals in low- and middle-income countries, leading to 2.6 million deaths^(3)^, and global medication errors cost an estimated $42 billion annually^(4)^. Therefore, the World Health Organization (WHO) has indicated that clear policies, organizational leadership capacity, data to drive safety improvements, skilled health care professionals and effective involvement of patients and families in the care process are all necessary to ensure sustainable and significant improvements in the safety of health care^(5)^.

Effective leadership is essential for the successful functioning of work, teams and the accomplishment of task goals^(6)^. Leadership is a process in which individuals use their leadership knowledge and skills to influence others in the organization to achieve common goals^(7)^. In 2010, the Institute of Medicine (IOM) reported “The Future of Nursing: Leading Change, Advancing Health”, challenging the nursing profession to enhance nursing’s leadership role in healthcare redesign^(8)^.

Nursing leadership plays a vital role in shaping the outcomes of healthcare organizations, personnel and patients^(9)^, especially in optimizing care and improving patient outcomes^(10)^. As a primary nursing manager, a head nurse directly leads frontline nurses to engage in clinical nursing work and is responsible for the quality of care and patient safety of each nursing unit^(11)^. The leadership of head nurses requires the ability to use their leadership knowledge, skills and attitudes to influence the attitudes, feelings and beliefs of others (service objects, colleagues or subordinates) in the organization and to urge them to take certain measures and behaviors to achieve common goals^(7)^. Nursing leadership is closely related to higher patient satisfaction and lower patient mortality, medication errors, restraint use and hospital-acquired infections^(12)^. While improving patient safety requires strong nursing leadership, nursing leadership does not directly affect patient safety outcomes but it does indirectly affect it through structural empowerment and staff nurses’ clinical leadership^(13)^. Furthermore, nursing leadership practices contribute to positive outcomes for nurses, including improved health and wellbeing, job satisfaction and retention^(14)^.

With the ever-changing and demanding healthcare environment, identifying and developing nursing leaders is one of the greatest challenges faced by the nursing profession^(15)^. However, targeted educational interventions have been identified as an effective method for improving nursing leadership^(9)^. Moreover, the new role described in the 2010 IOM report can be achieved through leadership programs focused on leadership training^(16)^.

However, currently, nursing leaders’ selection and appointment in China are based mainly on candidates’ clinical experience and expertise, and nursing leaders lack structured leadership training in human resources, conflict resolution, and quality and safety management, which significantly affects the scientific nature and effectiveness of the management of nursing leaders^(17)^. Therefore, a patient safety leadership program (PSLP) for head nurses was designed and conducted in a Chinese hospital. The objective of this study was to evaluate the impact of PSLP on head nurses and clinical nurses in the same nursing unit.

## Method

This pre-post quasi-experimental study was conducted ina grade A tertiary hospital with 1889 open beds and 60 nursing units in Changsha, Hunan Province, China. The head nurse of each nursing unit was invited to participate in the PSLP. If there was more than one head nurse in a nursing unit, the deputy head nurse was not invited to participate unless the head nurse did not meet the inclusion criteria. The inclusion criteria for head nurses were as follows: 1) currently serving as a head nurse; 2) being in the current position over 6 months; 3) not leaving current position within 12 months; and 4) not attending other similar training simultaneously.

Since the Hospital Leadership Behavior Assessment Scale (HLBAS) needs to be evaluated by several clinical nurses directly led by head nurses^(18-19)^, four clinical nurses (different levels of nurses, from N0 to N3) in the same nursing unit as each head nurse were recruited through a random number table to fill in the questionnaires anonymously before and after the training. The inclusion criteria for clinical nurses were as follows: 1) being in the current position over 6 months; 2) not leaving the current position within 12 months; and 3) not attending other studies simultaneously. Finally, a head nurse and four clinical nurses were recruited from each nursing unit, and a total of 60 head nurses and 240 clinical nurses participated in the study.

**Figure 1:**
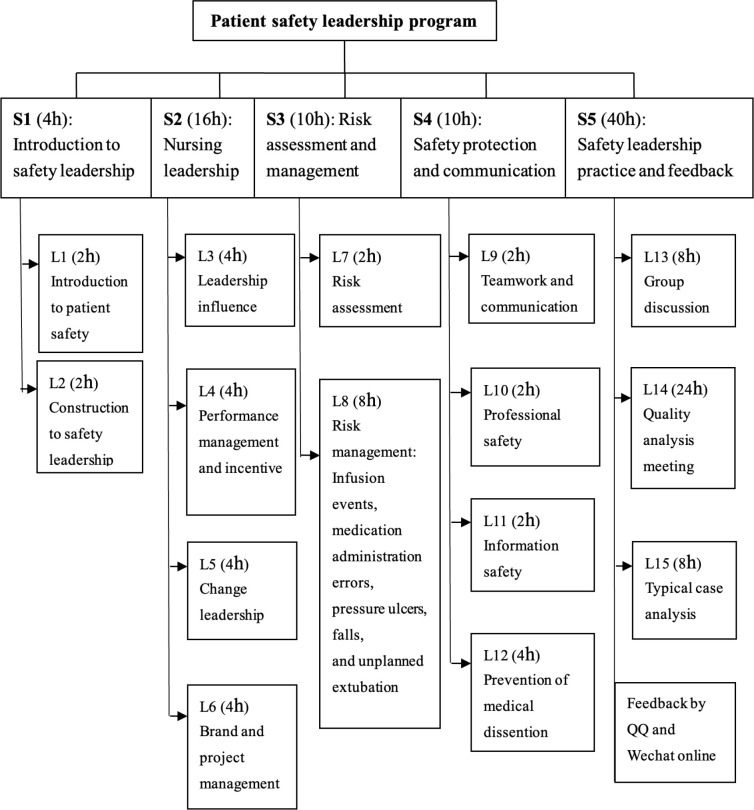
The schedule of PSLP. Changsha, Hunan Province, China, 2017-2018

**Figure 2:**
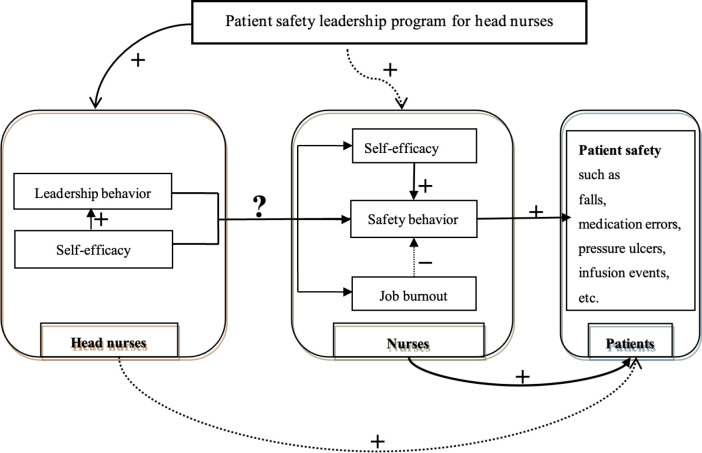
The effect model of the program. Changsha, Hunan Province, China, 2017-2018

The program started in May 2017 and ended in April 2018. All of the participants were informed of the details of the program by the staff in the nursing department through the hospital’s public mailbox. The content of the course was designed based on our previous studies^(20-21)^. The training involving 15 lessons requiring a total of 80 hours was divided into 5 sections. The details are shown in Figure 1, and the effect model is shown in Figure 2.

**Figure 3:**
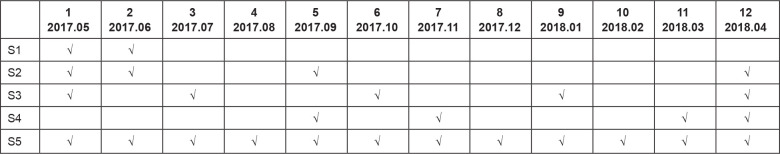
The timeline of the patient safety leadership program (PSLP). Changsha, Hunan Province, China, 2017-2018

With the aim of improving the knowledge professionalism, width and depth, a senior lecturer in MBA was invited to give the lessons for S2. Other theoretical lessons in S1 and S3 were given by clinical managers with rich experience in safety management, including directors and deputy directors of the nursing department, experts in safety management, and team leaders involved in hospital safety management. The feedback was conducted in S5 by QQ and WeChat online. The timeline of the PSLP is shown in Figure 3.

Four scales and a general information questionnaire were used in this study. The General Self-Efficacy Scale (GSES), the Hospital Leadership Behavior Assessment Scale (HLBAS), the Safety Behavior Scale (SBS), the Maslach Burnout Inventory (MBI) and the general information questionnaire were completed by the clinical nurses, while only the GSES and the general information questionnaire were completed by the head nurses. The first data collection was conducted a week before the first class started as a pre-test and the second data collection was conducted a week after the last class ended as a post-test.

The Chinese version of the GSES was developed to assess individuals’ self-efficacy with a Cronbach’s α of 0.87, retest reliability of 0.83, and split-half reliability of 0.82^(22-23)^. The GSES consists of 10 items, and each item is answered on a 4-point Likert scale ranging from one to four (“totally wrong” to “absolutely right”). A higher score represents higher self-efficacy.

The Chinese version of the MBI was developed with a Cronbach’s α of 0.93^(24)^. The MBI includes 3 dimensions with 22 items, and each item is answered on a 7-point Likert scale ranging from zero to six (“never” to “once a day”). A higher score indicates more severe burnout in the first two dimensions, while in the third dimension, the opposite is true.

The Chinese version of the SBS was introduced and revised with a Cronbach’s α of 0.91^(25)^. The scale consists of 12 items, and each item is answered on a 5-point Likert scale ranging from one to five (“never” to “always”). The scale was completed by the clinical nurses in our study to evaluate their safety behavior.

The HLBAS was translated and revised with a Cronbach’s α of 0.88^(26)^. The scale includes 2 dimensions with 20 items. The first 10 items evaluate the performance (P) function, which measures the head nurses’ efforts to improve the work efficiency and performance of the organization; the last 10 items evaluate the maintenance (M) function, which measures the head nurses’ efforts to coordinate and maintain the relationships within the organization^(18)^. Each item is answered on a 5-point Likert scale ranging from one to five (“totally inconsistent” to “absolutely consistent”). The score for the head nurses’ leadership behavior was the average of the four clinical nurses’ evaluation scores, and a higher score indicates better leadership behavior.

SPSS software version 23.0 (Armonk, New York) was used for data analysis. Absolute and relative frequencies, mean and standard deviation were used to present the descriptive statistics. The paired t-test was adopted to compare the scores of related variables of the four scales completed by clinical nurses or head nurses before and after the training. A value of P<0.05 was considered statistically significant.

This study was approved by the Ethics Committee of The Third Xiangya Hospital of Central South University (protocol number 2017-S027).

## Results

Sixty head nurses were recruited to participate in the PSLP training, and 240 clinical nurses were recruited to complete the four scales as the primary evaluation. All participants completed the entire process required for the study. The sociodemographic and professional characteristics of the participants are shown in Table 1. Most head nurses were aged 36-40 years old (51.7%, 31), and most clinical nurses were aged 31-35 years old (37.9%, 91).

**Table 1:** Sociodemographic and professional characteristics of clinical nurses and head nurses. Changsha, Hunan Province, China, 2017-2018 (CN^*^=240, HN^†^=60)

Characteristics	n^‡^(CN)	%^§^(CN)	n (HN)	% (HN)
**Department**				
Internal medicine	60	25,0	15	25,0
Surgery	80	33,3	20	33,3
Gynecology	12	5,0	3	5,0
Pediatrics	20	8,3	5	8,3
ICU	12	5,0	3	5,0
Operating room	4	1,7	1	1,7
Emergency	20	8,3	5	8,3
Others	32	13,3	8	13,3
**Sex**				
Male	8	3,3	1	1,7
Female	232	96,7	59	98,3
**Age**				
≤25	34	14,2	-	-
26-30	62	25,8	-	-
31-35	91	37,9	2	3,3
36-40	30	12,5	31	51,7
41-45	20	8,3	20	33,3
>45	3	1,3	7	11,7
**Years of nursing experience**				
≤5	66	27,5	-	-
6-10	72	30,0	-	-
11-15	78	32,5	23	38,3
16-20	24	10,0	34	56,7
>20	-	-	3	5,0
**Professional qualifications**				
Nurse	24	10,0	-	-
Primary nurse	94	39,2	-	-
Charge nurse	117	48,8	42	70,0
Associate professor or above	5	2,1	18	30,0
**First level of education**				
Technical secondary school	55	22,9	52	86,7
Junior college	112	46,7	-	-
Undergraduate	70	29,2	2	3,3
Master	3	1,3	5	8,3
Doctor	-	-	1	1,7
**Highest level of education**				
Technical secondary school	1	0,4	**-**	**-**
Junior college	15	6,3	2	3,3
Undergraduate	194	80,8	10	16,7
Master	30	12,5	46	76,7
Doctor	-	-	2	3,3
**Marital status**				
Married	175	72,9	54	90,0
Divorced	7	2,9	5	8,3
Unmarried	58	24,2	1	1,7
**Number of children**				
One	112	46,7	40	66,7
Two	52	21,7	19	31,7
None	76	31,7	1	1,7

* CN = Clinical nurse;

† HN = Head nurse;

‡ n = Number (absolute frequency);

§ % = Percentage

The mean scores of the GSES for the head nursesincreased significantly(p<0.01) from 2.95 (SD = 0.48) to 3.18 (SD = 0.41) compared with before the training. The total mean scores of the HLBAS for the head nurses increased significantly (p<0.001) from 75.38 (SD = 10.24) to 83.87 (SD = 7.66). Specifically, the mean score of the “performance” dimension increased significantly(p<0.001) from 37.57 (SD = 6.73) to 43.68 (SD = 4.79), and the mean score of the “maintenance” dimension increased significantly(p<0.05) from 37.82 (SD = 6.46) to 40.18 (SD = 5.76) after the training (Table 2).

The mean scores of the GSES for the clinical nurses increased significantly (p<0.001) compared with before the training from 2.71 (SD = 0.66) to 3.27 (SD = 0.63). The mean SBS scores of the clinical nurses increased significantly (p<0.001) from 3.66 (SD = 0.32) to 4.13 (SD = 0.36) after the training. The mean scores of the “emotional exhaustion” dimension decreased significantly (p<0.001) from 24.07 (SD = 9.46) to 20.51 (SD = 9.41) after the training. In contrast, the mean scores of the “personal accomplishment” dimension increased significantly (p<0.001) from 32.44 (SD = 7.65) to 39.54 (SD = 6.99) after the training (p<0.001). However, there was no significant difference for the mean scores of the “depersonalization” dimension (p = 0.140) (Table 2).

**Table 2:** Comparison of the scores of related variables between the clinical nurses and head nurses before and after the training. Changsha, Hunan Province, China, 2017-2018 (CN^*^=240, HN^†^=60)

Variables	Pretraining M^‡^(SD^§^)	Posttraining M(SD)	T	P-value^‖^
**GSES^¶^(HN)**	2.95(0.48)	3.18(0.41)	-3.03	<0.01
**GSES (CN)**	2.71(0.66)	3.27(0.63)	-9.76	<0.001
HLBAS**(HN)	75.38 (10.24)	83.87(7.66)	-5.14	<0.001
Performance (P)	37.57(6.73)	43.68(4.79)	-5.74	<0.001
Maintenance (M)	37.82(6.46)	40.18(5.76)	-2.12	<0.05
**SBS^††^(CN)**	3.66(0.32)	4.13(0.36)	-18.84	<0.001
**MBI^‡‡^(CN)**				
Emotional Exhaustion (EE)	24.07(9.46)	20.51(9.41)	4.25	<0.001
Depersonalization (DE)	7.75(6.47)	6.93(6.18)	1.48	0.140
Personal Accomplishment (PA)	32.44(7.65)	39.54(6.99)	-10.24	<0.001

* CN = Clinical nurse;

† HN = Head nurse;

‡ M = Mean;

§ SD = Standard Deviation;

|| P-value (Paired t-test);

¶ GSES = General Self-Efficacy Scale;

** HLBAS **=** Hospital Leadership Behavior Assessment Scale;

†† SBS = Safety Behavior Scale;

‡‡ MBI = Maslach Burnout Inventory

## Discussion

Few studies conducted to date have explored how to improve patient safety by enhancing the head nurses’ patient safety leadership, and few studies have used evaluation indexes of clinical nurses as the primary outcomes to verify the effectiveness of a training program. In this study, we designed and conducted a patient safety leadership program for head nurses and took clinical nurses’ self-efficacy, safe behavior and job burnout in the same nursing unit as the primary outcomes.

In this study, we observed that most head nurses who participated in the patient safety leadership program were under 40 years old, probably because the hospital we chose was only approximately 30 years old and the entire nursing team was relatively young. In this context, it is necessary to strengthen patient safety leadership training for head nurses.

Effective nursing leadership practices have a positive impact on nurses, health care quality and patient outcomes^(14,27-28)^. The results of this study showed that the patient safety leadership program for head nurses improved their self-efficacy and leadership behavior, promoted the clinical nurses’ safety behavior and self-efficacy, and reduced their job burnout.

Self-efficacy refers to a person’s belief in their ability to succeed in completing tasks or achieving goals, and the four sources of self-efficacy include direct experience, vicariousexperience, verbal persuasion and managing negative emotions^(29)^. Through the training, head nurses’ self-efficacy improved significantly. Perhaps the training program met the head nurses’ requirements for leadership change, risk assessment or management, safety protection, and teamwork or communication in patient safety management, which can provide successful experiences for their daily nursing safety management practices.

Likewise, this study discovered that head nurses’ leadership behavior improved significantly through the training. Head nurses’ transformation from knowledge and skills obtained in the training into nursing management practices was perceived by clinical nurses, which contributed to a significant improvement in the clinical nurses’ evaluation of the head nurses’ leadership behavior, including both the “performance” function and the “maintenance” function. Moreover, in terms of scores, the growth of the performance function was greater than that of the maintenance function. This implies that head nurses paid more attention to improving the organizational work performance, but they ignored maintaining relationships within organizations, possibly because the hospitals’ evaluation criteria only included nursing quality control and the incidence of nursing adverse events^(19)^.

Previous studies have confirmed that self-efficacy is an important factor affecting nursing leaders’ leadership behavior and that it can enhance nursing leaders’ leadership when applied strategically^(18,30)^. Regrettably, the relationship between the head nurses’ self-efficacy and leadership behavior was not explored further in our study.

Clinical nurses are caregivers whose safety behavior is closely related to patient safety^(31)^. This indicates that improvements in clinical nurses’ safety behavior contributes to enhancements of patient safety. A previous study found that head nurses’ leadership behavior has a positive effect on clinical nurses’ safety behavior, and safety culture perception is a mediating variable in the relationship between head nurses’ leadership behavior and clinical nurses’ safety behavior^(31)^. Consistent with this finding, the present study found that PSLP for head nurses significantly improved clinical nurses’ safety behavior in the same nursing unit. Therefore, hospital managers should cultivate head nurses’s leadership behavior, and head nurses should enhance self-cultivation, update nurses’ safety concepts, strengthen nurses’ theories, skills and other professional qualities, and urge nurses to develop safe nursing procedure behavior to further improve the nurses’ safety behavior.

Job burnout is a result of the interaction between a person and the work environment, which seriously affects employees’ work performance^(32)^. According to a cross-sectional survey of 2,504 nurses in eastern China, approximately 64.0% of nurses experienced job burnout that led to a lower efficiency and quality of work^(33)^. However, it has been reported that there is a significant negative correlation between head nurses’ leadership and clinical nurses’ job burnout^(34)^. This indicates that the better the head nurses’ leadership, the lower the clinical nurses’ burnout. Our study found that clinical nurses’ “emotional exhaustion” was significantly reduced and clinical nurses’ “personal accomplishment” was significantly increased. Head nurses may reduce clinical nurses’ work pressure and workload, improve their organizational identity, and finally reduce their job burnout by creating a good work environment and providing appropriate support^(35)^. However, clinical nurses’ “depersonalization” was not promoted significantly in our study. This may be due to the features of the course content in this training program, which mainly promoted the skills of risk assessment, prevention and coping with adverse events, teamwork and communication in patient safety management but did not emphasize the importance of human care. As a consequence, head nurses did not pay more attention to the improvement of clinical nurses’ human care.

Furthermore, self-efficacy, leadership behavior, safety behavior and job burnout influence each other. For head nurses, self-efficacy has critical implications for the improvement of leadership^(18,30)^. For clinical nurses, higher self-efficacy means better nursing behavior and a higher quality of care^(36)^. Clinical nurses’ job burnout is associated with worse patient safety outcomes, such as medical errors^(37)^. Nevertheless, self-efficacy can adjust and balance clinical nurses’ job burnout. Therefore, this is a complex process. Nursing leaders’ leadership behaviors, especially transformational leadership behaviors, contribute to creating workplace conditions that promote better safety outcomes for nurses and patients^(38-39)^. In the present study, the PSLP for head nurses did not benefit clinical nurses and patients directly but positively influenced clinical nurses indirectly by creating a good patient safety culture atmosphere. Finally, clinical nurses may positively influence patient outcomes by exhibiting good leadership skills.

Our previous studies on patient safety ignored clinical nurses’ psychosocial indexes even though they are closely related to patient safety^(20-21)^, so the effect of the PSLP for head nurses was evaluated mainly from the perspective of clinical nurses. The above positive results indicate that the program worked well for head nurses and that what they learned was incorporated into their safety management practices. Thus, nursing leaders should attach importance to the patient safety leadership training of head nurses, adopt a mixed training approach and make detailed plans for the training after a comprehensive consideration of the training objectives, the content practicability and its difficulty. In the PSLP, we adopted a variety of training methods, such as theory teaching, group discussion, quality analysis meetings, typical case analysis and online feedback. Recently, experiential training (including experience, sharing, communication, integration and application) with good practicability has been proven to be effective in improving head nurses’ leadership and personal qualities in enhancing their participation and enthusiasm^(40)^. Therefore, we need innovation for the training methods used to improve nursing leadership.

There were several limitations in this study. First, the sample size was small, and only one hospital was involved. Second, the lack of a control group made it impossible tocompare the effect of the PSLP with that of the traditional training program. Finally, evaluation indexes from patients were not included. A large-sample, multicenter randomized controlled trial will be conducted, and the effect of the intervention will be evaluated from the perspective of patients in the future.

## Conclusion

In conclusion, the patient safety leadership program had a positive impact on the self-efficacy and leadership behavior of head nurses, including their performance function and their maintenance function. Moreover, the safety behavior, self-efficacy and job burnout of clinical nurses improved after the training. Overall, the training program for head nurses can benefit not only head nurses but also clinical nurses. This will ultimately improve the quality of care and the patient safety outcomes in each nursing care unit.
